# Catalysts for implementation of One Health in Kenya

**DOI:** 10.11604/pamj.supp.2017.28.1.13275

**Published:** 2017-11-02

**Authors:** Athman Mwatondo, Peninah Munyua, Zeinab Gura, Mathew Muturi, Eric Osoro, Mark Obonyo, Austine Bitek, Harry Oyas, Murithi Mbabu, Jackson Kioko, Kariuki Njenga, Sara Lowther, Samuel Mwangi Thumbi

**Affiliations:** 1 Zoonotic Disease Unit, Ministry of Health, Nairobi, Kenya; 2 Division of Global Health Protection, US Centers for Disease Control and Prevention, Nairobi, Kenya; 3 Field Epidemiology and Laboratory Training Program, Ministry of Health, Nairobi, Kenya; 4 Zoonotic Disease Unit, Ministry of Agriculture, Livestock and Fisheries, Nairobi, Kenya; 5 Veterinary Epidemiology and Economics Unit, Ministry of Agriculture, Livestock and Fisheries, Nairobi, Kenya; 6 Disease Surveillance, Vector and Zoological Services, Ministry of Agriculture, Livestock and Fisheries, Nairobi, Kenya; 7 Department of Preventive and Promotive Health Services, Ministry of Health, Nairobi, Kenya; 8 Paul G. Allen School for Global Animal Health, Washington State University, Pullman, WA, USA; 9 Centers for Global Health Research, Kenya Medical Research Institute, Kenya

**Keywords:** One health, Global Health, preparedness, public health events, Africa

## Abstract

The recent Zika outbreak in the Americas, Ebola epidemic in West Africa and the increased frequency and impact of emerging and re-emerging infections of animal origin have increased the calls for greater preparedness in early detection and responses to public health events. One-Health approaches that emphasize collaborations between human health, animal health and environmental health sectors for the prevention, early detection and response to disease outbreaks have been hailed as a key strategy. Here we highlight three main efforts that have progressed the implementation of One Health in Kenya.

## Commentary

The recent Zika outbreak in the Americas, Ebola epidemic in West Africa and the increased frequency and impact of emerging and re-emerging infections of animal origin have increased the calls for greater preparedness in early detection and responses to public health events [1-4]. Given global integration and the ease and speed of travel between countries and continents, these recent epidemics have demonstrated that a health threat anywhere can easily turn to a threat everywhere. One-Health approaches that emphasize collaborations between human health, animal health and environmental health sectors for the prevention, early detection and response to disease outbreaks have been hailed as a key strategy [5]. Whereas the concept of One-Health has been largely accepted across the developed and developing world and among international human health and animal health organizations, its implementation has faced challenges. These challenges are mainly driven by separate institutional and policy mandates whereby public health departments work to secure human health, while animal health departments efforts focus to promote trade and mitigate the huge economic consequences that arise from animal diseases. Other challenges include the historical “silo” mentality in human and animal health training in managing human and animal health threats, lack of guidance on how to operationalize the One-Health approach at national and international levels and weak capacity for surveillance and response in the animal health sector when compared to the human health sector [6,7]. Despite similar challenges, Kenya has in the last 7 years made significant progress in implementing One-Health. Focus on three main efforts have catalyzed this progress.

The first was the establishment of a national One-Health office, referred to as the Zoonotic Disease Unit (ZDU), to coordinate surveillance and outbreak response activities between Kenya’s Ministries responsible for Human Health and Animal Health [8]. The ZDU, established in 2012, has provided Kenya with an institutional framework that allows zoonotic diseases to receive dedicated attention. This is important given zoonotic diseases in Kenya, like in many countries, are neglected in light of other health priorities. The ZDU has created unique opportunities for tackling zoonoses including incorporation of zoonotic diseases reporting into the health ministry integrated disease surveillance and response framework, disease specific surveillance among animal and human populations for MERS-CoV and anthrax, the development of Kenya’s first strategic plan for implementation of One-Health, prevention and control plans such as the Rift Valley fever integrated preparedness and response plan that aims to enhance the country’s capacity to mitigate adverse impact of future outbreaks and a rabies elimination plan that aims to eliminate human rabies by 2030 [9]. Through the ZDU, Kenya was able to determine the zoonotic diseases of national importance as well as develop an evidence-based prioritization of these diseases for informing decision-making on their prevention and control [10]. Institutionalization of One Health through the ZDU has also created a forum for communications and sharing of data between the human and animal health ministries and among research partners working on zoonosis.

The second main contributor to the success of One Health in Kenya has been the continued development of a One-Health workforce within the country. The Field Epidemiology and Laboratory Training Program (FELTP) that provides Masters level training in applied epidemiology to veterinarians and medical doctors working within government ministries has played a significant role. Although more than 60% of the infectious diseases in humans and 75% of emerging and re-emerging infections, are of animal origin [11], veterinarians and physicians are schooled separately and work in different institutions with different mandates, creating distinct boundaries between the professions and little opportunities for collaboration. The FELTP training in Kenya, which began in 2004, allows each FELTP resident to lead an outbreak investigation including from zoonotic diseases as part of their training requirement. This joint training approach has begun to break down professional boundaries and created linkages and networks between the two professions thereby enhancing collaboration. The effectiveness of a similar FELTP training was well demonstrated in Nigeria in 2015, where presence of a functional emergency operation center (created for polio eradication) and the coordination role by the Nigeria FELTP graduates working within government service were thought to be two of the main reasons why Nigeria was able to contain the Ebola incursion, preventing the epidemic from spreading [1].

As of 2016, the Kenya FELTP has completed training for 135 medical and 15 veterinary epidemiologists, with many of the graduates actively serving in the public sector. These epidemiologists have enhanced the capacity of the human and animal health sectors in disease surveillance, outbreak response and improved collaborations between the human and animal health sectors. As many of these officers assume middle-level positions in their respective ministries, their appreciation and embrace of the One-Health approach has contributed significantly to the practice of One-Health in the face of major public health threats. The ZDU effectiveness has also been enhanced by the fact that the medical and veterinary epidemiologists working at the Unit have been jointly trained and understand the workings of each other´s ministries well. In addition to FELTP, there have been efforts to revise the training curricula in the medical, veterinary and public health schools to include One-Health approaches. The curriculum revision effort has been led by the United States Agency for International Development’s workforce development program referred to as One-Health Central and Eastern Africa working in 7 countries within sub-Saharan Africa; Kenya, Uganda, Tanzania, Rwanda, Ethiopia, Cameroon and Democratic Republic of Congo.

The third main contributor to the success of One Health implementation in Kenya has been the active and close associations maintained between the ZDU and multilateral and bilateral agencies and partners, local and international research institutions and universities working on zoonosis in Kenya. Through this multi-sectoral coordination role, ZDU has advocated for and coordinated funding for key One Health projects. These close relationships are enhanced through regular quarterly Zoonotic Technical Working Group meetings and specific disease research programs such as those on the burden and transmission of brucellosis and operational research on rabies elimination. These programs have enhanced the translation of research findings into public health intervention actions. A good example is the elevation of risk of RVF outbreak associated with the 2015/16 El Niño rains, during which the government of Kenya was able to use data from research by ZDU and partners to provide a predictive RVF risk-map so that they could institute a targeted surveillance and response for the disease during the high risk period [12].

The World Health Organization’s (WHO) International Health Regulations (IHR), the World Organization for Animal Health (OIE) Performance of Veterinary Services and the more recent Global Health Security Agenda are all aimed at supporting countries to prepare, detect and respond to health threats and all recognize the critical role that One Health collaborations play. While implementation of the One Health approach at national level is notable, there remain significant gaps in its implementation at subnational administrative levels under the devolved governance adopted in Kenya in 2013. Veterinary and health services including surveillance and laboratory diagnosis for zoonoses have been devolved to 47 counties, thereby creating a necessity to also devolve the One Health coordination mechanisms. This is currently a focus for implementation of One Health in Kenya in both creating One-Health coordination units and improving surveillance and response to outbreaks at the county level.

The challenges associated with enhancing this approach practically within countries remain daunting. These challenges include competing priorities, funding deficiencies and breaking a silo mentality within and between human and animal professions. It is, however, our opinion that three specific efforts in Kenya; the establishment of the ZDU, One-Health workforce development including joint trainings for Veterinarians and Physicians through the FELTP program and the deliberate efforts to foster collaboration between institutions working on zoonosis have not only significantly accelerated the uptake and practice of One Health in Kenya, but have contributed to increased visibility of zoonotic diseases in Kenya and improvement of Kenya’s preparedness against health threats of animal origin.

Disclaimer: the findings and conclusions in this report are those of the authors and do not necessarily represent the official position of the U.S. Centers for Disease Control and Prevention.

## Competing interests

The authors declare no competing interest.
